# DDESC: Dragon database for exploration of sodium channels in human

**DOI:** 10.1186/1471-2164-9-622

**Published:** 2008-12-20

**Authors:** Sunil Sagar, Mandeep Kaur, Adam Dawe, Sundararajan Vijayaraghava Seshadri, Alan Christoffels, Ulf Schaefer, Aleksandar Radovanovic, Vladimir B Bajic

**Affiliations:** 1 South African National Bioinformatics Institute, University of the Western Cape, Private Bag- X17, Modderdam Road, Bellville, Cape Town 7535, South Africa

## Abstract

**Background:**

Sodium channels are heteromultimeric, integral membrane proteins that belong to a superfamily of ion channels. The mutations in genes encoding for sodium channel proteins have been linked with several inherited genetic disorders such as febrile epilepsy, Brugada syndrome, ventricular fibrillation, long QT syndrome, or channelopathy associated insensitivity to pain. In spite of these significant effects that sodium channel proteins/genes could have on human health, there is no publicly available resource focused on sodium channels that would support exploration of the sodium channel related information.

**Results:**

We report here Dragon Database for Exploration of Sodium Channels in Human (DDESC), which provides comprehensive information related to sodium channels regarding different entities, such as "genes and proteins", "metabolites and enzymes", "toxins", "chemicals with pharmacological effects", "disease concepts", "human anatomy", "pathways and pathway reactions" and their potential links. DDESC is compiled based on text- and data-mining. It allows users to explore potential associations between different entities related to sodium channels in human, as well as to automatically generate novel hypotheses.

**Conclusion:**

DDESC is first publicly available resource where the information related to sodium channels in human can be explored at different levels. This database is freely accessible for academic and non-profit users via the worldwide web .

## Background

Sodium channels are heteromultimeric, integral membrane proteins that conduct the sodium ions (Na^+^) through plasma membrane of the cell. The classification of sodium channels is based on the trigger that opens the channel for ions, i.e. voltage-gated sodium channels (triggered by a voltage-change) and ligand-gated sodium channels (triggered by binding of a ligand to the channel) [1]. The mutations in genes coding for sodium channel proteins have been linked with several genetic disorders, called 'sodium channelopathies' such as inherited febrile epilepsy, autism, Brugada syndrome, ventricular fibrillation, long QT syndrome, etc [2-7]. Recently, *SCN9A *gene which encodes for *NaV1.7 *voltage-gated sodium channel, has been linked to molecular pathophysiologies of pain disorders like inherited erythromelalgia and inherited paroxysmal extreme pain disorder (PEPD) and has emerged as a therapeutic target for treatment of neuropathic pain [8] Additionally, nearly 20 disorders affecting skeletal muscle contraction, cardiac rhythm, or neuronal function have been linked to these mutations in human. It has also been shown that sodium channel mutations could cause alteration in the physiological properties (hyperexcitability or hypoexcitability) of the cells depending upon which sodium channels genes are expressed. Both sodium channel mutations and cell background contribute to neuronal function and clinical manifestations [9].

Due to the complexity of molecular functioning and effects that sodium channels have, it is important for biologists and medical researchers to have means to explore the relevant information in an easy fashion. However, the information regarding the sodium channels is scattered through the literature or across various public and commercial databases. To the best of our knowledge there is no resource focused specifically to sodium channels, though there are two publicly accessible ion channel databases: the Ion Channel Database  and International Union of Pharmacology database [10]. These databases provide mainly sequence information about the genes encoding for different ion channels. Consequently, there is a need for a focused comprehensive public resource that allows users to explore information related to sodium channels from multiple angles.

As the amount of scientific literature continues to increase, text-mining is becoming more important in extracting and summarizing information from the literature. Text-mining also ensures the investigation of latest and wider range of publications. We present here Dragon Database for Exploration of Sodium Channels in Human (DDESC), a sodium channel biology resource, compiled based on text- and data-mining. It provides comprehensive information about genes and proteins, metabolites and enzymes, toxins, chemicals with pharmacological effect, disease concepts, human anatomy, pathways and pathway reactions, potentially associated with sodium channel, and provides potential links between these entities. The present study introduces a database for exploring human sodium channels in order to provide useful information for drug development. Various computational approaches, such as structural bioinformatics [11-14], molecular docking [15-20], pharmacophore modelling [21], QSAR [22-27], protein sub-cellular location prediction [28,29], identification of membrane proteins and their types [30], identification of enzymes and their functional classes [31], identification of proteases and their types [32], protein cleavage site prediction [33-35], and signal peptide prediction [36,37], provide useful information and insights for both basic research and drug design. All these fields of research can further benefit from DDESC and hence the database can serve a wider science community. The database is accessible via the worldwide web , where it will be regularly updated. The access is free for academic and non-profit users.

## Construction

DDESC is generated by the licensed OrionCell's  Dragon Exploration System (DES) tool. DES uses dictionary based text-mining approach for extracting potentially relevant information from text documents. The functioning of the text-mining modules of DDESC is based on similar concepts as used in [38] and [39]. DES has previously been utilized in the creation of a part of the DDOC database [40]. In our study, DES is applied with six OrionCell's proprietary and manually curated dictionaries for "human genes and proteins", "metabolites and enzymes", "toxins", "chemical with pharmacological effects", "disease concepts" and "human anatomy". The dictionaries contain numerous variants of names and symbols customary for the specific types of entities. For example the dictionary of genes and proteins contains over 300,000 variants of entities covering the names, symbols, aliases, previous names and previously used symbols of genes and proteins, compiled from the literature and published databases.

The information in DDESC was based on 5,243 abstracts retrieved from PubMed repository  on 2008-08-06 by using the query ("sodium channel" OR "sodium channels") human. DES then maps all the entities from the dictionaries to the documents submitted for the analysis and the extracted information is then compiled into a database. DDESC provides the summarized lists of entities, frequency of published documents, frequency of pairs of entities, as well as clustering of documents based on entities found. The details on how to use the database and other relevant details about the methods applied by DES are provided in the documentation .

## Utility

**Figure 1:**
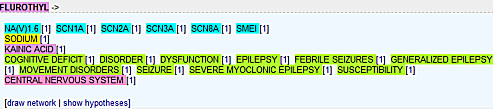
**Potential association of ‘FLUROTHYL’ a ‘Chemical with pharmacological effects’ with other entities**. Clicking on the numbers next to the entities will open the relevant abstract(s).

To date, there is no resource available, which could provide detailed information about the various potential biological interactions related to sodium channels. The DDESC is the first publicly available resource where the user can explore multiple information regarding sodium channels at molecular, chemical and functional level (Fig 1). The information can be further curated and analysed in a constructive and systematic way. The database provides a user friendly interface in an easy to follow color-coding and graphical representations.

All the entities in DDESC are further linked to the literature through the PubMed IDs. Each entity is also linked to Reactome pathways, as well as associated chemical reactions within the pathways. Another useful aspect of DDESC is its ability to generate association hypotheses for further investigation. User can generate the hypotheses by selecting any combination of the used dictionaries (see documentation). Associations between the different entities can also be viewed as a network. DDESC also provides search options by using simple logical operators i.e. "AND", "OR" and "NOT" that will further allow the users for easier and more direct access to each of the reports.

## Discussion

DDESC is the first text- and data-mining based integrated knowledgebase that allows researchers to get an overview and explore efficiently the biology of sodium channels. For text-mining, a dictionary based method is used where comprehensive list of genes and other entities are matched against the documents for entity recognition and information extraction. If an entity is found, it is tagged as originating from the dictionary that is being processed at that moment. Due to the sequential processing of the dictionaries, it is not possible to tag already tagged entity again, even if such an entity exists in the other, not yet processed, dictionaries. Once all dictionaries are processed, all analysed PubMed abstracts are annotated by the entities from dictionaries as identified in the text. However the main difficulty in entity recognition is the lack of standardisation of the name. Each gene and protein has several synonyms and abbreviations, some of which are common English words. Entity association is based on the co-occurrence of the entities within the abstracts or sentences. If the two entities are repeatedly mentioned together, there are chances that they are linked directly or indirectly. The users could further explore such associations in the context of sodium channels by, for example, inspecting the documents from which the association was extracted. DDESC gives the user flexibility to change the reference dictionary to explore different type of entity associations (see documentation for details). Networks can be generated for the different types of entity associations, which can further be zoomed into the chosen terms and relations by expanding the correlation tree and selecting the preferred subsets of dictionaries. Networks help in bringing different types of data at one platform for its better understanding.

**Figure 2:**
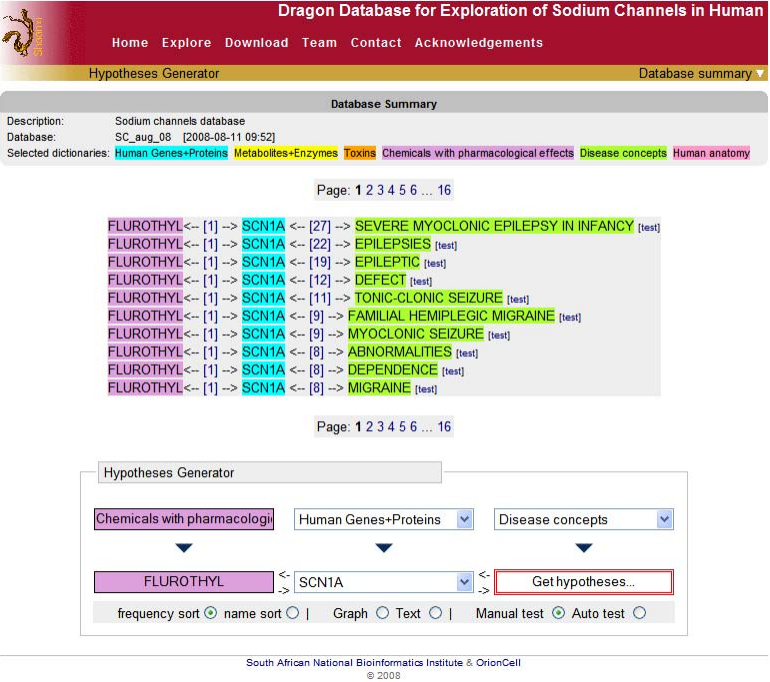
**Example of Hypotheses generated by using ‘FLUROTHYL’ under the dictionary ‘Chemicals with pharmacological effects’ and ‘SCN1A’ gene under the dictionary ‘Human Genes and proteins’**. At the time of writing this publication the ‘test’ link between ‘FLUROTHYL’ and ‘EPILEPSIES’ retrieved eight PubMed documents.

Hypothesis generation is another useful feature, which allows users to infer new relationships for different entities (Fig 2). The idea for the hypothesis generation is that if entity A is linked to entity B and entity B is further linked to entity C then there may be a new relationship between entity A and entity C. Manually, it is very difficult to infer new hypothesis based on all the published facts, specially with the vast amount of available literature. Clicking on the 'test' link, one can further inspect validity of the hypothesis generated in DDESC, by retrieving the PubMed document(s) related to the entities linked through the hypothesis. If no such PubMed document is found, this could suggest a possible genuine new hypothesis for further exploration.

Evaluation of the accuracy of integrated data is a necessary step and generally is expressed in terms of precision (the ability to identify the correct entities in the abstracts relative to the number of all identified entities of the same type), recall (the ability to identify correct entities of specific type present in the abstracts relative to all such entities present in the abstracts) and F-measure [41]. Since it is not possible to evaluate every concept from each of the dictionaries across all 5,243 documents, we selected the *SCN1A *gene as the reference. The *SCN1A *gene is one of the most studied sodium channel genes and more than 100 mutations have been identified within over 100 kb of its exon harbouring regions. *SCN1A *is also clinically very relevant as it contributes to the largest number of mutations linked with epilepsy [42]. For this purpose, we manually curated all 131 abstracts in which *SCN1A *gene has been explicitly identified. List of abstracts can be easily obtained on page 'summary of links'.

The analysis of the results for *SCN1A *gene shows that the precision and recall are in the range of 81%–100% (Table 1) depending on the type of dictionary used. Overall, DDESC exploration database system has efficiently identified most of the entities from the 131 abstracts related to the *SCN1A *gene with an average F-measure value of 92.9%. One should note, however, that these estimated quality parameters are only for the *SCN1A *gene and are based on 131 abstracts. The results may vary for the other entities. It is shown in Table 1 that 81.1% of the entities related to genes and proteins are correctly identified while 14 entities (SCN, SCN1, Potassium channel, VOLTAGE-GATED K+ CHANNEL, VOLTAGE-GATED POTASSIUM CHANNEL, GTP, Parvalbumin, P17, P21, PL-3, AED, GEF and SMEI) were wrongly identified as genes or proteins. This is due to the fact that some of these entities refer to the family of genes (for example VOLTAGE-GATED POTASSIUM CHANNEL, *SCN*, *CYP*), some entities are recognized partly (for example, *SCN1 *which is a part of *SCN1-3A*) and others are the synonyms of genes and proteins that are used as abbreviations for other biological entities in the abstracts. For example, SMEI is one of the aliases for *SCN1A *gene (sodium channel, voltage-gated, type I, alpha) and has been placed in the list of genes and proteins. However, in the relevant text SMEI actually referred to the term 'Severe myoclonic epilepsy of infancy' (PubMed ID: 11359211). Similarly *GEF *is a synonym for *ARHGEF2 *gene (rho/rac guanine nucleotide exchange factor (GEF) 2), but it is also an acronym for generalized epilepsy with febrile seizures plus (GEFS+).

**Table 1:** Precision, recall and F-measure of entity recognition in documents related to SCN1A gene

Dictionaries used	Number of entities identified	Number of correctly identified entities	Precision (%)	Recall (%)	F-measure (%)
Genes and proteins	74	60	81.1	96.1	87.9

Metabolites and enzymes	28	28	100.0	100.0	100.0

Chemicals with pharmacological effects	7	6	85.7	100.0	92.3

In DDESC, more than 96% of genes and proteins symbols mentioned in 131 *SCN1A*-related abstracts were identified. The exceptions are *FHM1*, *Na(v)1.7 *and *Na(V)1.1 *and these are synonyms for *CACNA1A*, *SCN9A *and *SCN1A *genes respectively. In the list of identified chemicals with pharmacological effects, the only wrong term is 'lead', which is a metal and metabolite, but also a very common English word. Such entities contribute to lowering the precision. This is one of issues in the DES tool, which will require further improvements.

To check the efficiency of the system in comparison to others, we further did a brief comparison with recently published tool, PolySearch [43] The reported PolySearch F-measure for gene synonym identification, protein-protein interaction identification and disease gene identification is 88%, 81% and 79%, respectively. We submitted the '*SCN1A*' gene as a query to PolySearch and searched for metabolites and drugs related to *SCN1A *gene mentioned in these abstracts. PolySearch identified only six entities in total whereas DDESC has provided 28 metabolites and enzymes including drugs for *SCN1A *gene. The comparison in the category of genes and proteins shows that DDESC identified 31 more entities as compared to PolySearch, which identified 14 of them. This difference could be partially due to the content of the dictionaries. For example, PolySearch has total approximately 234,000 entities for genes and proteins and their synonyms in their dictionary whereas DES contains over 300,000 entities for genes and proteins and their synonyms and other names. Detailed comparison of genes and proteins results can be found in Additional file 1.

We also looked if the entities identified actually do relate to sodium channels. Through manual curation of 131 abstracts, we identified entities directly associated with the functionality of sodium channels [see Additional file 1]. In the category of "genes and proteins", 18 (30%) out of total 60 entities were found to be either genes coding for various sodium channel proteins, or genes or proteins that could directly affect the functionality of sodium channel proteins. In the list of metabolites and enzymes for *SCN1A *gene, 19 (68%) entities out of total 28 were found to be directly associated with sodium channels. There were three (50%) out of six chemicals with pharmacological effects that affect the functionality of sodium channels. The "disease concepts" are too broad to be linked directly to sodium channels. Rather these could be linked more appropriately with a specific disease and in the case of *SCN1A *gene, it is epilepsy.

## Future directions

In future, as the list of the entities will grow and will be further curated, the improvements in the quality of dictionaries will certainly enhance the accuracy of the database. The comments obtained from the users will also help to incorporate changes that will make DDESC more useful. We also plan to include additional functionality like search options and batch query into the database in future revisions.

## Conclusion

DDESC is aimed to serve as one-stop data warehouse for sodium channel research. The database could be of interest to researchers in different areas of biomedical and pharmaceutical research to identify targeted literature and possible associations/interactions between biological entities relevant to sodium channels. We hope that this database will serve as a useful complement to the existing public resources as being specialized for sodium channel biology. We also hope that the ability to generate association hypotheses could lead to new scientific concepts for further exploration. DDESC will be updated every six months and the information from all new studies published in that period will be incorporated.

## Abbreviations

DDESC: Dragon Database for Exploration of Sodium Channels in Human; DES: Dragon Exploration System.

## Competing interests

VBB and AR are partners in the OrionCell company whose product, Dragon Exploration System, has been used in creation of DDESC. Other authors declare no conflict of interest.

## Authors' contributions

SS, MK and VBB conceptualized the study, analyzed results and wrote the manuscript. SS, MK, SVS, AD, AC and US performed the analysis. AR and VBB developed the DES system.

## Supplementary Material

Additional file 1 Comparison of genes and proteins results with other systems. Detailed comparison of the results for genes and proteins identified by PolySearch and DDESC along with the detailed list of 45 DDESC genes. Click here for file

## References

[B1] Catterall WA (2000). From ionic currents to molecular mechanisms: the structure and function of voltage-gated sodium channels. Neuron.

[B2] Bezzina C, Veldkamp MW, Berg MP van Den, Postma AV, Rook MB, Viersma JW (1999). A single Na(+) channel mutation causing both long-QT and Brugada syndromes. Circ Res.

[B3] Chen Q, Kirsch GE, Zhang D, Brugada R, Brugada J, Brugada P (1998). Genetic basis and molecular mechanism for idiopathic ventricular fibrillation. Nature.

[B4] George AL (2005). Inherited disorders of voltage-gated sodium channels. J Clin Invest.

[B5] Miller TM, Dias da Silva MR, Miller HA, Kwiecinski H, Mendell JR, Tawil R (2004). Correlating phenotype and genotype in the periodic paralyses. Neurology.

[B6] Wang Q, Shen J, Li Z, Timothy K, Vincent GM, Priori SG (1995). Cardiac sodium channel mutations in patients with long QT syndrome, an inherited cardiac arrhythmia. Hum Mol Genet.

[B7] Weiss LA, Escayg A, Kearney JA, Trudeau M, MacDonald BT, Mori M (2003). Sodium channels SCN1A, SCN2A and SCN3A in familial autism. Mol Psychiatry.

[B8] Dib-Hajj SD, Cummins TR, Black JA, Waxman SG (2007). From genes to pain: Na v 1.7 and human pain disorders. Trends Neurosci.

[B9] Waxman SG (2007). Channel, neuronal and clinical function in sodium channelopathies: from genotype to phenotype. Nat Neurosci.

[B10] Catterall WA, Goldin AL, Waxman SG (2003). International Union of Pharmacology. XXXIX. Compendium of voltage-gated ion channels: sodium channels. Pharmacol Rev.

[B11] Chou KC (2004). Structural bioinformatics and its impact to biomedical science. Curr Med Chem.

[B12] Chou KC (2004). Insights from modeling three-dimensional structures of the human potassium and sodium channels. J Proteome Res.

[B13] Chou KC (2004). Modelling extracellular domains of GABA-A receptors: subtypes 1, 2, 3, and 5. Biochem Biophys Res Commun.

[B14] Schnell JR, Chou JJ (2008). Structure and mechanism of the M2 proton channel of influenza A virus. Nature.

[B15] Chou KC, Wei DQ, Zhong WZ (2003). Binding mechanism of coronavirus main proteinase with ligands and its implication to drug design against SARS. Biochem Biophys Res Commun.

[B16] Gao WN, Wei DQ, Li Y, Gao H, Xu WR, Li AX (2007). Agaritine and its derivatives are potential inhibitors against HIV proteases. Med Chem.

[B17] Li Y, Wei DQ, Gao WN, Gao H, Liu BN, Huang CJ (2007). Computational approach to drug design for oxazolidinones as antibacterial agents. Med Chem.

[B18] Wang JF, Wei DQ, Chen C, Li Y, Chou KC (2008). Molecular modeling of two CYP2C19 SNPs and its implications for personalized drug design. Protein Pept Lett.

[B19] Zhang R, Wei DQ, Du QS, Chou KC (2006). Molecular modeling studies of peptide drug candidates against SARS. Med Chem.

[B20] Zheng H, Wei DQ, Zhang R, Wang C, Wei H, Chou KC (2007). Screening for new agonists against Alzheimer's disease. Med Chem.

[B21] Sirois S, Wei DQ, Du Q, Chou KC (2004). Virtual screening for SARS-CoV protease based on KZ7088 pharmacophore points. J Chem Inf Comput Sci.

[B22] Dea-Ayuela MA, Perez-Castillo Y, Meneses-Marcel A, Ubeira FM, Bolas-Fernandez F, Chou KC (2008). HP-Lattice QSAR for dynein proteins: experimental proteomics (2D-electrophoresis, mass spectrometry) and theoretic study of a Leishmania infantum sequence. Bioorg Med Chem.

[B23] Du Q, Mezey PG, Chou KC (2005). Heuristic molecular lipophilicity potential (HMLP): a 2D-QSAR study to LADH of molecular family pyrazole and derivatives. J Comput Chem.

[B24] Du QS, Huang RB, Chou KC (2008). Recent advances in QSAR and their applications in predicting the activities of chemical molecules, peptides and proteins for drug design. Curr Protein Pept Sci.

[B25] Du QS, Huang RB, Wei YT, Du LQ, Chou KC (2008). Multiple field three dimensional quantitative structure-activity relationship (MF-3D-QSAR). J Comput Chem.

[B26] Gonzalez-Diaz H, Gonzalez-Diaz Y, Santana L, Ubeira FM, Uriarte E (2008). Proteomics, networks and connectivity indices. Proteomics.

[B27] Prado-Prado FJ, Gonzalez-Diaz H, de la Vega OM, Ubeira FM, Chou KC (2008). Unified QSAR approach to antimicrobials. Part 3: first multi-tasking QSAR model for input-coded prediction, structural back-projection, and complex networks clustering of antiprotozoal compounds. Bioorg Med Chem.

[B28] Chou KC, Shen HB (2007). Recent progress in protein subcellular location prediction. Anal Biochem.

[B29] Chou KC, Shen HB (2008). Cell-PLoc: a package of Web servers for predicting subcellular localization of proteins in various organisms. Nat Protoc.

[B30] Chou KC, Shen HB (2007). MemType-2L: a web server for predicting membrane proteins and their types by incorporating evolution information through Pse-PSSM. Biochem Biophys Res Commun.

[B31] Shen HB, Chou KC (2007). EzyPred: a top-down approach for predicting enzyme functional classes and subclasses. Biochem Biophys Res Commun.

[B32] Chou KC, Shen HB (2008). ProtIdent: a web server for identifying proteases and their types by fusing functional domain and sequential evolution information. Biochem Biophys Res Commun.

[B33] Chou KC (1993). A vectorized sequence-coupling model for predicting HIV protease cleavage sites in proteins. J Biol Chem.

[B34] Chou KC (1996). Prediction of human immunodeficiency virus protease cleavage sites in proteins. Anal Biochem.

[B35] Shen HB, Chou KC (2008). HIVcleave: a web-server for predicting human immunodeficiency virus protease cleavage sites in proteins. Anal Biochem.

[B36] Chou KC, Shen HB (2007). Signal-CF: a subsite-coupled and window-fusing approach for predicting signal peptides. Biochem Biophys Res Commun.

[B37] Shen HB, Chou KC (2007). Signal-3L: A 3-layer approach for predicting signal peptides. Biochem Biophys Res Commun.

[B38] Pan H, Zuo L, Choudhary V, Zhang Z, Leow SH, Chong FT (2004). Dragon TF Association Miner: a system for exploring transcription factor associations through text-mining. Nucleic Acids Res.

[B39] Bajic VB, Veronika M, Veladandi PS, Meka A, Heng MW, Rajaraman K (2005). Dragon Plant Biology Explorer. A text-mining tool for integrating associations between genetic and biochemical entities with genome annotation and biochemical terms lists. Plant Physiol.

[B40] Kaur M, Radovanovic A, Essack M, Schaefer U, Maqungo M, Kibler T (2008). Database for exploration of functional context of genes implicated in ovarian cancer. Nucleic Acids Res.

[B41] Malik R, Franke L, Siebes A (2006). Combination of text-mining algorithms increases the performance. Bioinformatics.

[B42] Mulley JC, Scheffer IE, Petrou S, Dibbens LM, Berkovic SF, Harkin LA (2005). SCN1A mutations and epilepsy. Hum Mutat.

[B43] Cheng D, Knox C, Young N, Stothard P, Damaraju S, Wishart DS (2008). PolySearch: a web-based text mining system for extracting relationships between human diseases, genes, mutations, drugs and metabolites. Nucleic Acids Res.

