# Supplementary material for: DDESC: Dragon database for exploration of sodium channels in human
Source: BMC Genomics. 2008 Dec 20;9:622. doi: 10.1186/1471-2164-9-622 (PMC2631582; doi:10.1186/1471-2164-9-622)
Supplement: Additional file 1 — Comparison of genes and proteins results with other systems. Detailed comparison of the results for genes and proteins identified by PolySearch and DDESC along with the detailed list of 45 DDESC genes. [file 1471-2164-9-622-S1.doc]

**Additional file for**

Sunil Sagar, Mandeep Kaur, Adam Dawe, Sundararajan Vijayaraghava Seshadri, Alan Christoffels, Ulf Schaefer, Aleksandar Radovanovic, Vladimir B. Bajic **DDESC: Dragon Database for Exploration of Sodium Channels in Human**

Comparison of the results for ‘genes and proteins’ identified by PolySearch and DDESC is presented here (Additional Table 1). In PolySearch, total 159 entities were found by using query “SCN1A”, which includes a list of 20 genes and their synonyms. During the hand curation of the data, we found 20 duplicates and 5 ambiguous entities (not genes or proteins). The number of true genes was found to be 14 with 120 synonyms.

In DDESC, 74 entries are linked to “SCN1A” gene. Fourteen ambiguous entries and 15 synonyms were found after manual curation. Therefore, 45 were the true entries for genes and proteins. Out of these 45, 14 entries are common with PolySearch. This concludes that DDESC has found all the entities searched by PolySearch plus 31 new entries.

**Additional Table 1 Comparison of results between PolySearch and DDESC for genes and proteins**

|  | PolySearch | DDESC |
| --- | --- | --- |
| Total entities identified | 139 (after removing 20 duplicates) | 74 |
| Ambiguous entries | 5 | 14 |
| Synonyms | 120 | 15 |
| True entries | 14 | 45 |
| Common between PolySearch and DDESC | 14 | 14 |
| Entries found by DDESC but not by PolySearch | NA | 31 |
| Entries found by PolySearch but not by DDESC | 0 | NA |

The detailed list of 45 DDESC genes is presented in the Additional Table 2. All these 45 genes are found in DDESC whereas only 14 genes are found by PolySearch. The commonly found genes/proteins are colored in red.

**Additional Table 2 New genes or proteins in DDESC as well as genes or proteins common to PolySearch**

| Gene or protein | DDESC | PolySearch |
| --- | --- | --- |
| Feb1 |  |  |
| Feb2 |  |  |
| Feb5 |  |  |
| Feb6 |  |  |
| ATPASE |  |  |
| CACNA1A |  |  |
| CACNB4 |  |  |
| CALMODULIN |  |  |
| CHRNA4 |  |  |
| CHRNB |  |  |
| CHRNB2 |  |  |
| CLCN2 |  |  |
| COMT |  |  |
| CYP2C9 |  |  |
| CYP3A5 |  |  |
| EFHC1 |  |  |
| FHM2 |  |  |
| FHM3 |  |  |
| GABRA1 |  |  |
| GABRD |  |  |
| GABRG2 |  |  |
| GCH1 |  |  |
| GM3 SYNTHASE |  |  |
| HIRIP5 |  |  |
| KCNA1 |  |  |
| KCNQ2 |  |  |
| KCNQ3 |  |  |
| LAFORIN |  |  |
| LGI1 |  |  |
| MAPT |  |  |
| MASS1 |  |  |
| NHLRC1 |  |  |
| NIFU |  |  |
| NOVA2 |  |  |
| P-GLYCOPROTEIN |  |  |
| SCN11A |  |  |
| SCN1B |  |  |
| SCN2A1 |  |  |
| SCN2B |  |  |
| SCN3A |  |  |
| SCN4A |  |  |
| SCN5A |  |  |
| SCN7A |  |  |
| SCN8A |  |  |
| SCN9A |  |  |

**Additional Table 3 List of entities linked with sodium channel biology based on 131 abstracts**

| Gene or protein | Metabolites | Chemical with Pharmacological effects |
| --- | --- | --- |
| CALMODULIN | Aspartate | Aspartic acid |
| NOVA2 | Decarboxynucleic acid | Flurothryl |
| SCN11A | Glutamine | Kainic acid |
| SCN1B | Glycine |  |
| SCN2A1 | Histidine |  |
| SCN2B | Luciferase |  |
| SCN3A | Arginine |  |
| SCN4A | Oxcarbazepine |  |
| SCN5A | Carbamazepine |  |
| SCN7A | Valproate |  |
| SCN8A | Topiramate |  |
| SCN9A | PHT |  |
| SCN1A | Sodium ion |  |
| Nav1.1 | Threonine |  |
| Nav1.2 | Triphosphatase |  |
| Nav1.3 | Lysine |  |
| Nav1.6 | Calcium |  |
| PN1 | Sodium |  |
|  | Phenytoin |  |
